# Supplementary material for: Crystal scavenging from mush piles recorded by melt inclusions
Source: Nat Commun. 2019 Dec 20;10:5797. doi: 10.1038/s41467-019-13518-2 (PMC6925248; doi:10.1038/s41467-019-13518-2)
Supplement: Supplementary file 2 — Supplementary Information [file 41467_2019_13518_MOESM2_ESM.pdf]

Supplementary Information: Crystal scavenging from mush piles recorded by melt inclusions

Penny E. Wieser[*1], Marie Edmonds[1], John Maclennan[1], Frances Jenner[2] and Barbara E. Kunz[2].

[1] *Department of Earth Sciences, University of Cambridge, Downing Street, Cambridge, UK, CB2 3EQ.*

[2]*School of Environment, Earth and Ecosystem Sciences, The Open University, Walton Hall, 7 Milton Keynes, Buckinghamshire, MK7 6AA.*

**Corresponding Author: pew26@cam.ac.uk*

Additional Figures

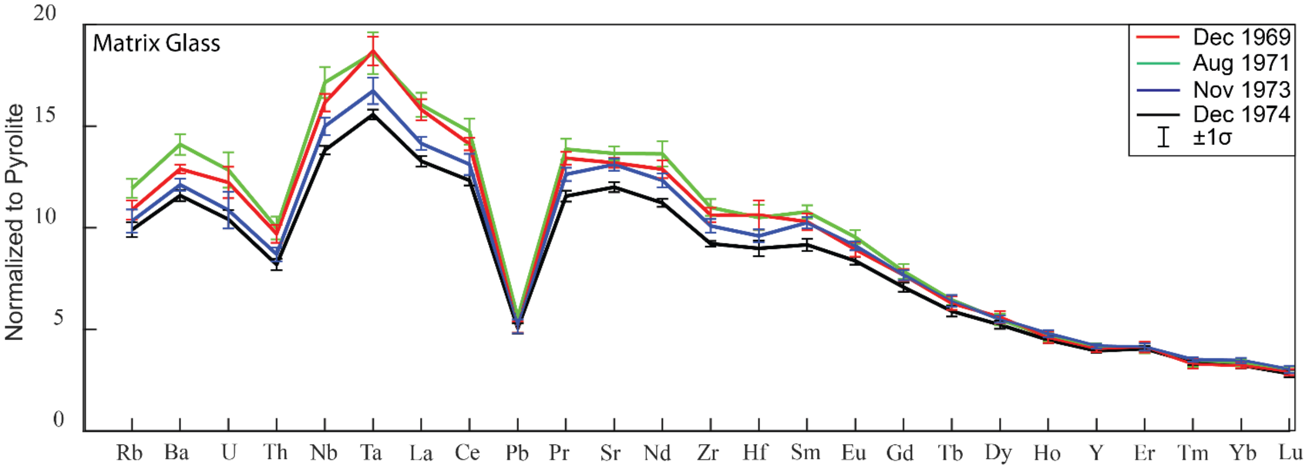

Supplementary Figure 1- a) Spider diagram of matrix glass compositions from the four eruptions considered in this study. The mean composition is shown, with error bars ±1σ of repeated analyses. There are clear variations in chemistry with time exceeding analytical error. Normalized to Pyrolite (additional ref.[1]).

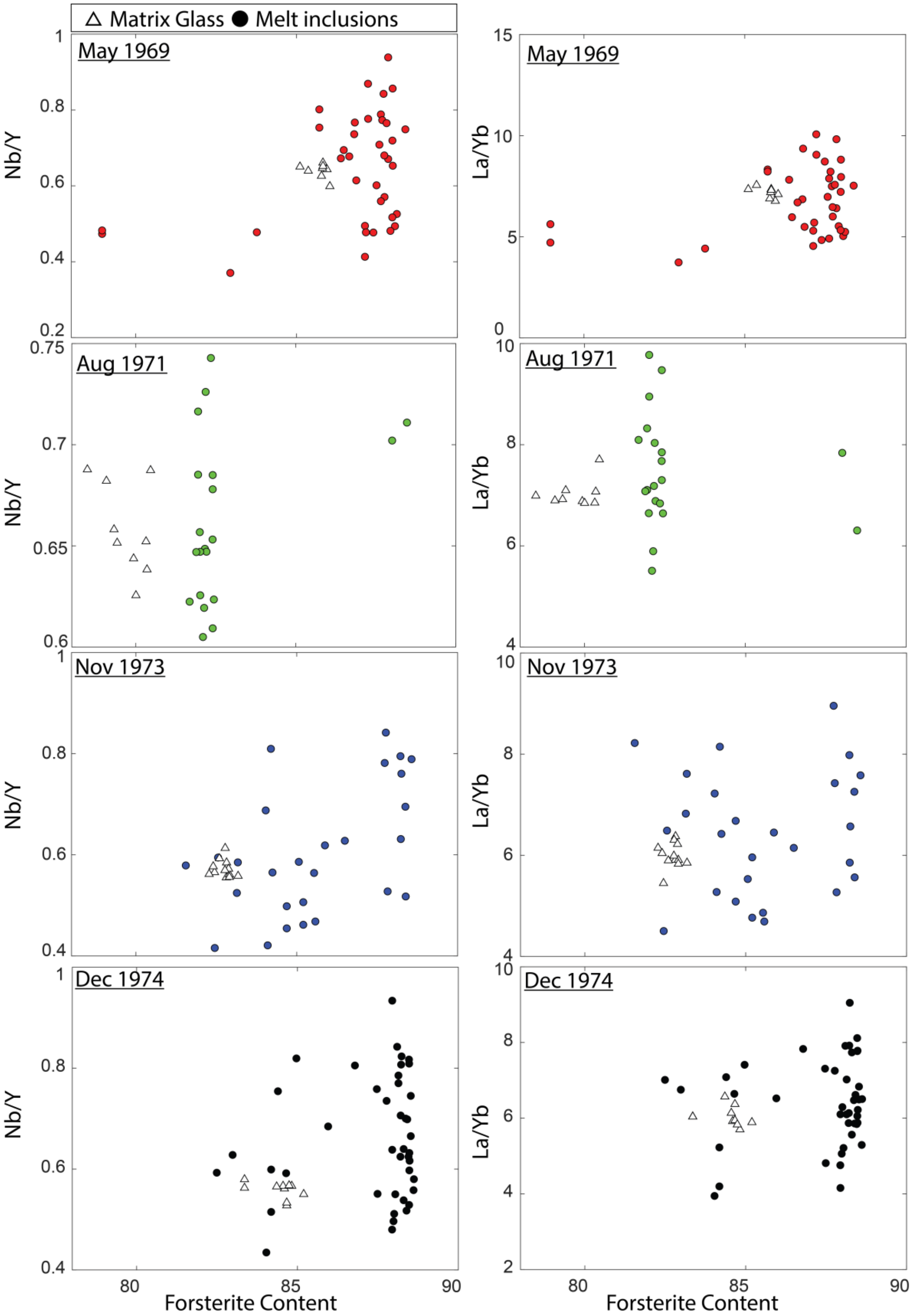

Supplementary Figure 2- Trace element ratios versus forsterite content for each eruption. The equilibrium olivine composition for glass was calculated for K_D=0.3, Fe^{3+}/Fe_T=0.15 (~QFM). Unlike icelandic eruptions, there is no obvious decline in trace element diversity with decreasing forsterite content[2,46].

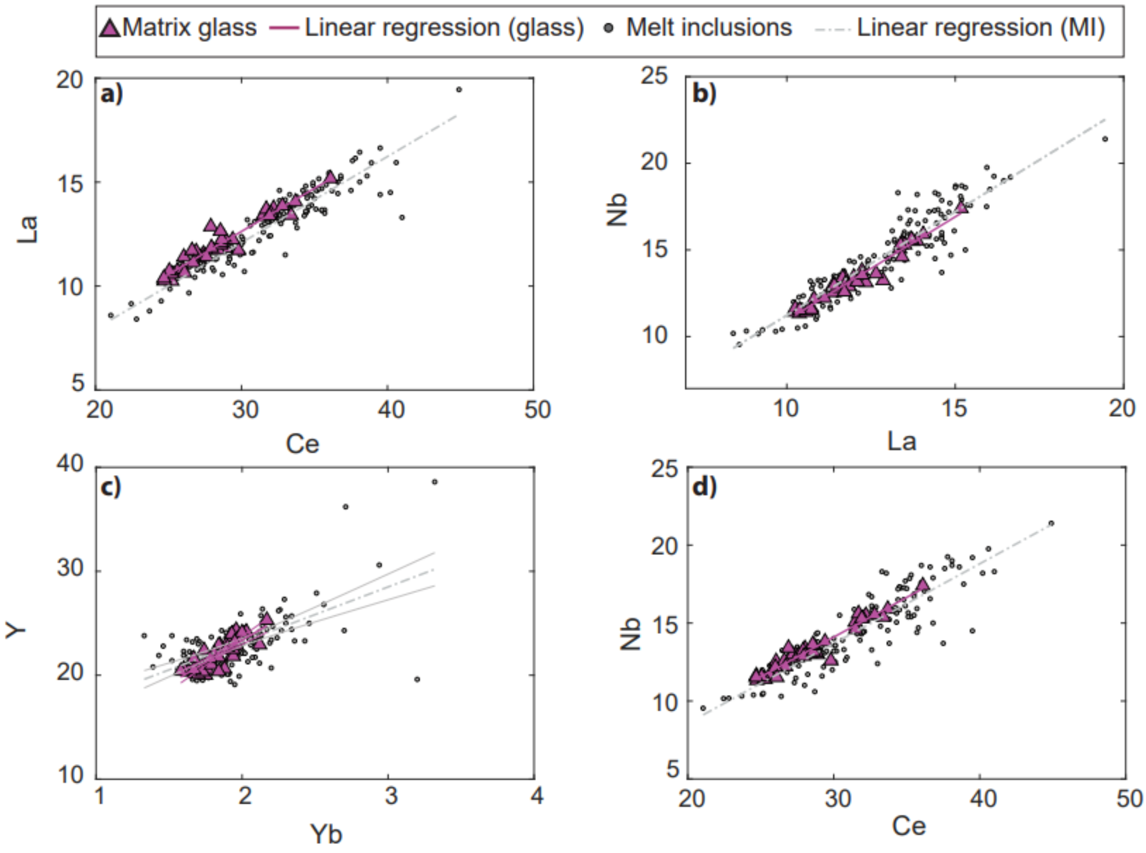

Supplementary Figure 3-Matrix glasses and melt inclusions show strong positive correlations between incompatible elements (e.g. La vs. Ce), due to variations in the extent of mantle melting. If trace elements were undergoing diffusional re-equilibration in melt inclusions, the regression lines defined by the melt inclusions (grey lines) and the glasses (pink lines) would deviate from one another in b-d). This is not observed. 95% confidence intervals are shown for c), as the relatively compatibility of these elements produces weaker correlations.

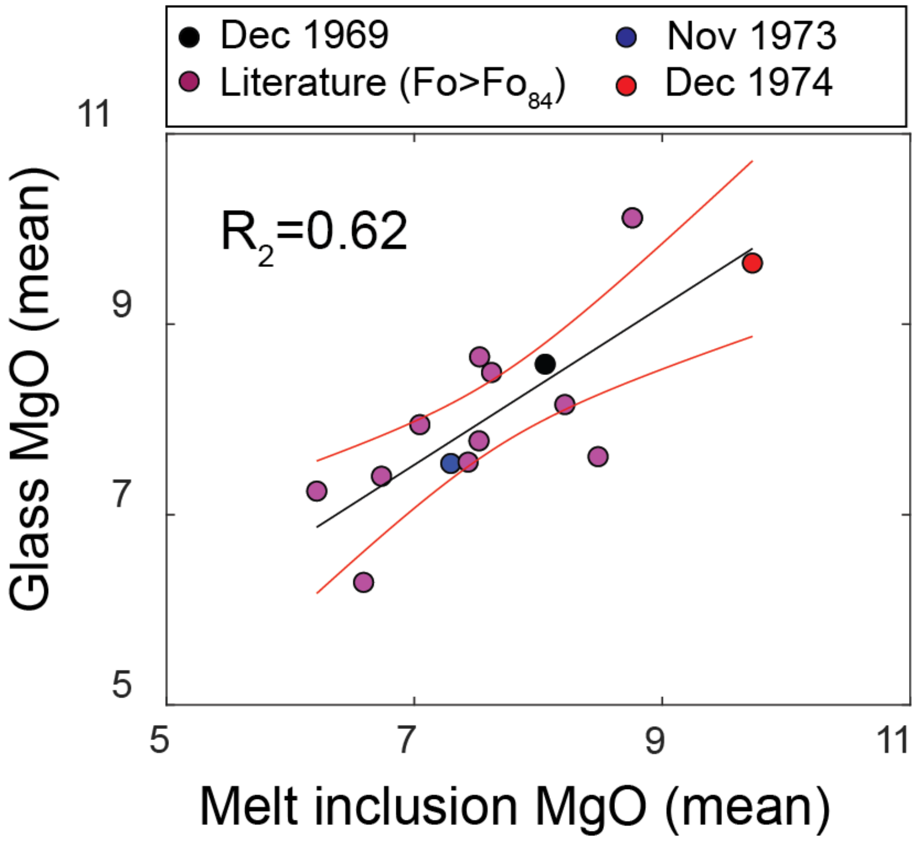

Supplementary Figure 4- There is a strong correlation between melt inclusion and matrix glass MgO contents for eruptions with $\overline{\mathrm{Fo}} > \mathrm{Fo}_{84}$. This demonstrates the rapidity of post-entrapment crystallization following crystal scavenging.

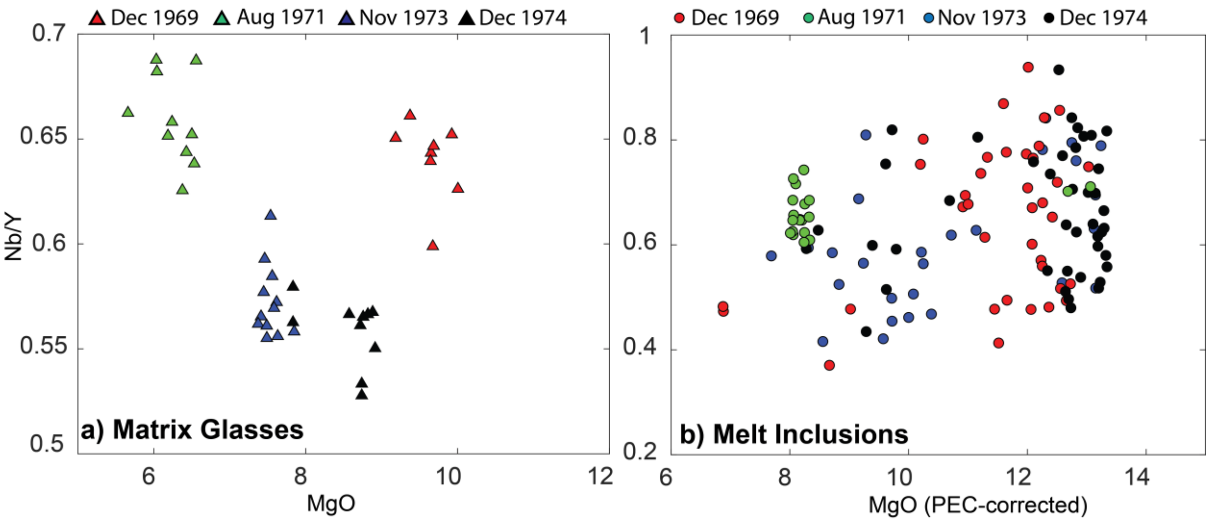

Supplementary Figure 5- Nb/Y vs. MgO in matrix glasses (a) and PEC-corrected MgO in melt inclusions (b). There is no correlation between Nb/Y and MgO, supporting our statements in the main text that Nb/Y ratios are largely unaffected by olivine and chromite fractionation.

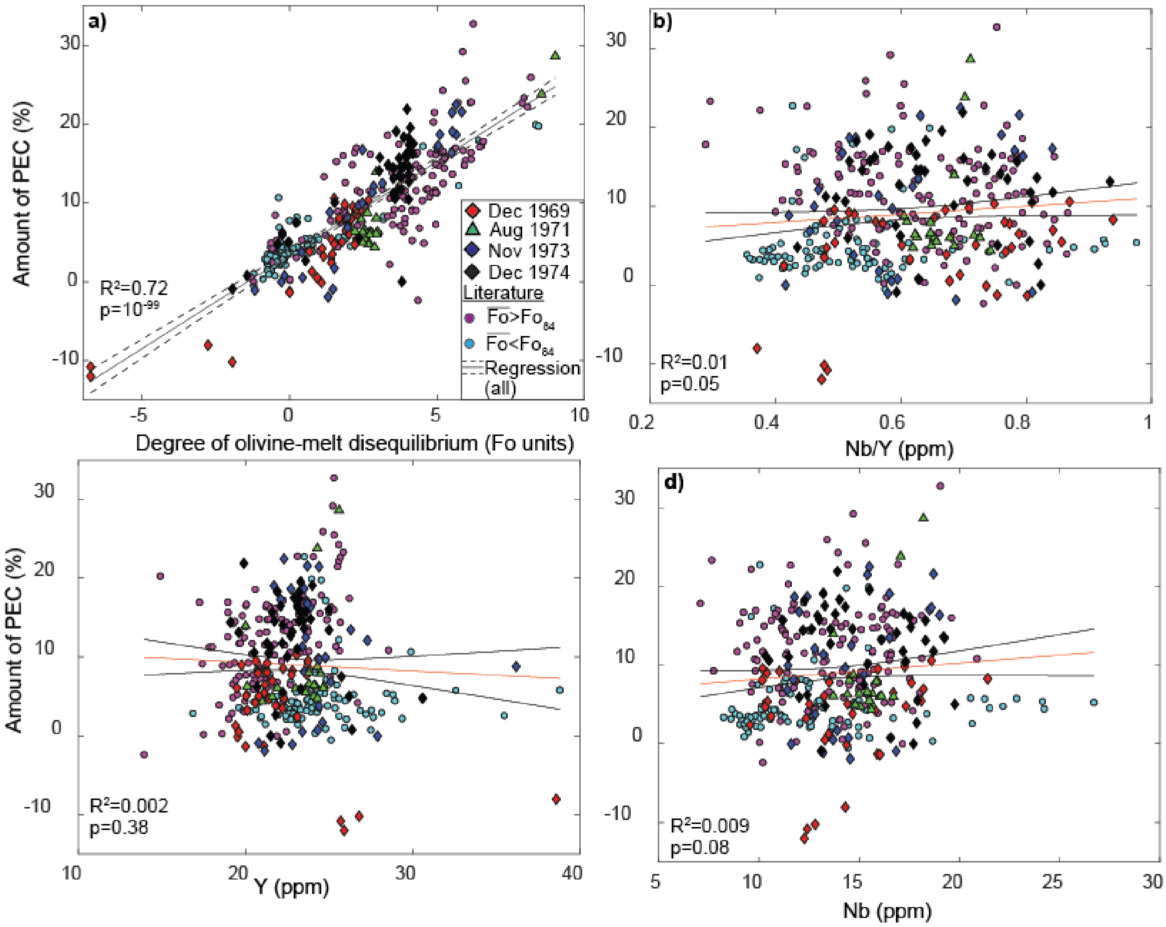

Supplementary Figure 6- Nb and Y systematics versus the amount of PEC. Figure 4a from the main text is shown for context in **a**). **b-d)** There is no correlation between the amount of PEC and Nb/Y ratios or Nb and Y concentrations. This demonstrates that Nb/Y is largely unaffected by PEC.

Additional Tables

Supplementary Table 1- Calibration materials, count times, and estimates of analytical precision and accuracy for EPMA analysis of olivines hosting melt inclusions. Precision and accuracy was estimated from repeated measurements of a San Carlos Olivine secondary standard.

Element	Calibration Material	Crystal	Peak Count Time	Condition	Order	Precision (%) (Std dev/ mean)	Accuracy (%) (measured/ standard)
Si	Diopside	TAP	10s	1	1st	0.74	100.2
Mg	St. Johns Olivine	TAP	20s	1	2nd	0.53	99.8
Fe	Fayalite	LIF	20s	1	1st	1.02	99.8
Al	Corundum	LTAP	120s	2	1st	7.68	97.9

Ti	Rutile	PET	120s	2	1st	76.24	132.7
Ca	Diopside	LPET	10s	2	1st	6.23	89.5
Ni	Nickel oxide	LIF	60s	2	1st	3.13	102.1
Cr	Cr metal	LIF	30s	2	2nd	48.73	118.1
Mn	Mn metal	LIF	30s	2	3rd	8.41	100.3

Supplementary Table 2- Calibration materials and count times for EPMA analyses of matrix glasses and melt inclusions.

**Element**	**Calibration Material**	**Crystal**	**Peak Count Time**	**Condition**	**Analysis order**
Na	Jadeite	LTAP	10s	1	1st
Al	Corundum	LTAP	30s	1	2nd
P	Apatite	PET	30s	1	1st
Ca	Diopside	PET	30s	1	2nd
K	K feldspar	LPET	10s	1	1st
Ti	Rutile	LPET	60s	1	2nd
Si	Diopside	TAP	10s	1	1st
Mg	St. Johns Olivine	TAP	30s	1	2nd
Fe	Fayalite	LIF	20s	1	1st
Mn	Manganese	LIF	40s	1	2nd
S	Pyrite	PET, LPET	60s, 60s	2	1st
Cl	Halite	PET, LPET	60s, 60s	2	2nd

Supplementary Table 3- Precision and accuracy for EPMA analyses of matrix glasses and melt inclusions. Precision and accuracy was estimated from repeated measurements of VG2 and A99 secondary standards. Cl and MnO concentrations were consistently higher than long-term laboratory averages. Their concentrations were divided by 1.15 and 1.65 respectively to correct for this offset.

**Element**	**A99 Precision (%) (Std dev/mean)**	**A99 Accuracy (%) (measured/standard)**	**VG2 Precision (%) (Std dev/mean)**	**VG2 Accuracy (%) (measured/standard)**
Na_2O	6.21	95.6	2.71	98.2
Al_2O_3	0.90	98.7	0.60	98.1
P_2O_5	6.93	97.6	17.76	92.8
K_2O	0.91	101.2	0.99	101.2
CaO	2.30	99.6	9.39	99.2
TiO_2	0.56	100.6	0.83	99.3
SiO_2	0.48	98.6	0.58	99.3
MgO	2.25	99.9	2.26	101.1
FeO	1.12	98.8	1.21	99.2
MnO	10.22	159.4	5.54	175.1
SO_2	18.50	91.1	1.83	98.0
Cl	16.48	115.8	6.84	112.9

Olivine Forsterite Compilation

Olivine compositions shown in Fig.3b include 689 core analysis from a wide variety of eruptions located across the Kīlauean edifice. * indicates eruptions chosen for melt inclusion analysis. This compilation excludes the forsterite contents of olivines hosting melt inclusions, in case the forsterite distribution of these were skewed.

Supplementary Table 4- Detailed information on the different eruptions for which olivine core analyses were performed in this study.

**Sample**	**Eruption Date**	**Description**	**Location**	**GPS co-ordinates**
KL0905	July 19-22, 1974	Lua Manu Fissure	Extracaldera	19˚ 23.933‘ N, 155˚ 15.337‘ W
KL0909	May 24th, 1969	Ep. 1 Mauna Ulu	ERZ	19˚ 21.826‘ N, 155˚ 12.877‘ W
KL0908*	Dec 30th, 1969	Ep. 12 Mauna Ulu	ERZ	19˚ 20.839‘ N, 155˚ 12.518‘ W
KL0910*	Nov 10th, 1973	Pau'ahi Crater	ERZ	19˚ 22.313‘ N, 155˚ 13.510‘ W
KL0914	Sept 24-29th, 1971		Intracaldera	19˚ 24.580‘ N, 155˚ 16.631‘ W

KL0916*	Aug 14th, 1971		Intracaldera	19˚ 24.137‘ N, 155˚ 16.644‘ W
KL0917	Late July, 1974		Intracaldera	19˚ 24.06‘ N, 155˚ 16.653‘ W
KL0919*	Late Dec, 1974	Ka’u Desert	SSWRZ	19˚ 22.649‘ N, 155˚ 17.609‘ W
KL0920	Late Dec, 1974	Ka’u Desert	SSWRZ	19˚ 22.603‘ N, 155˚ 17.713‘ W
KL0922	Sept 1974		Intracaldera	19˚ 24.201’ N, 155˚ 17.502’ W
KL0924	Late July, 1974		Extracaldera	19˚ 24.142’ N, 155˚ 16.896’ W
KL0930	1919-1920	Mauna Iki	NSWRZ	19˚ 21.230’ N, 155˚ 23.892’ W

Supplementary Table 5-Analytical conditions for 3 separate EPMA sessions used to produce olivine Fo content database used for Fig. 3b. Some moderate instrument drift occurred in session 2. Repeated analyses of San Carlos olivine were used to create a linear interpolation model to correct for this drift.

**Element**	**Calibration**	**Crystal**	**Peak Count time**	**Analysis order**	**Condition**	**Precision (%) (Std dev/ mean)**	**Accuracy (%) (measured/ standard)**
Session 1 – 30 nA, 15 kV. November, 2017							
Si	Diopside	TAP	10s	1st	N/A	0.49	99.0
Mg	St. Johns Olivine	TAP	20s	2nd	N/A	1.16	97.7
Fe	Fayalite	LIF	20s	1st	N/A	1.07	99.3
Session 1 – February 2018 – Dual condition run<br>Cond1 = 30 nA, 15 kV; Cond 2 = 100 nA, 15 kV							
Si	Diopside	TAP	10s	1st	1	1.12	99.26
Mg	St. Johns Olivine	TAP	20s	2nd	1	2.41	100.30
Fe	Fayalite	LIF	20s	1st	1	1.14	98.47
Session 3 –40 nA, 15 kV. March 2018,							
Si	Diopside	TAP	20s	1st	N/A	0.67	100.7
Mg	St. Johns Olivine	LTAP	20s	1st	N/A	0.72	99.9
Fe	Fayalite	LIF	20s	1st	N/A	0.93	100.58

Supplementary Table 6- Percent variation for summit whole-rock compositions reported in ref. 36. The percent variation was calculated in two ways:

$$\textit{Percent Variation (range)} = 100 \text{ x} \frac{Range\ (1790-1982)}{Mean\ (1790-1982)}$$

$$\textit{Percent Variation } (1\sigma) = 100 \text{ x} \frac{1\sigma\ (1790-1982)}{Mean\ (1790-1982)}$$

	**Nb/Y**	**Zr/Y**	**La/Y**	**La/Yb**	**Sm/Yb**	**La/Sm**	**Ba/Nb**
Percent Variation $(range)_{1790\text{-}1982}$	57.1	23.8	54.27	55.5	22.7	38.2	18.7
Percent Variation $(1\sigma)_{1790\text{-}1982}$	15.7	6.6	13.8	13.8	5.8	9.0	5.2
Accuracy (measured/preferred BCR-2G value)	0.998	0.997	1.00	0.990	0.977	1.02	1.00
Precision (std dev/mean)	5.23	3.33	4.71	4.45	5.83	6.56	5.68

Supplementary References

1. McDonough, W. & Sun, S. The composition of the Earth. *Chemical Geology* **120**, 223–253 (1995).
