# Supplementary material for: Crystal scavenging from mush piles recorded by melt inclusions
Source: Nat Commun. 2019 Dec 20;10:5797. doi: 10.1038/s41467-019-13518-2 (PMC6925248; doi:10.1038/s41467-019-13518-2)
Supplement: Supplementary file 3 — Description of Additional Supplementary Files [file 41467_2019_13518_MOESM3_ESM.pdf]

Description of Additional Supplementary Files

File Name: Supplementary Data 1
Description: Excel spreadsheet of major and trace element concentrations in melt inclusions and glasses, and olivine major element data reported in this study.

File Name: Supplementary Data 2
Description: Forsterite contents compiled from the literature, and analysed for this study.
